# Prevalence and Severity of Sleep Bruxism in Edentulous Patients: A Cross-Sectional Study

**DOI:** 10.1155/2024/7498654

**Published:** 2024-11-08

**Authors:** Naiara Luchi Klöppel, Patrícia Pauletto, Naiany Meiriely de Almeida Lopes, Franciele Floriani, Rangel Lidani, Graziela De Luca Canto, Analucia Gebler Philippi, Luis André Mezzomo

**Affiliations:** ^1^Private Practice, Brazil; ^2^Faculty of Dentistry, Universidad De Las Américas, Quito, Ecuador; ^3^University of Iowa at Prosthodontics Department, Iowa City, Iowa, USA; ^4^Department of Dentistry, Federal University of Santa Catarina, Florianópolis, Brazil; ^5^Department of Restorative Dentistry, University of Illinois Chicago, Chicago, Illinois, USA

**Keywords:** edentulous jaw, electromyography, prevalence, sleep bruxism

## Abstract

**Aims:** Edentulous patients can also suffer from sleep bruxism (SB), just like dentate patients. This study aims to evaluate the prevalence and severity of SB in edentulous patients using the definitive method.

**Methods:** Twenty-three edentulous patients underwent treatment with new conventional complete dentures. Definitive SB was evaluated using the Bruxoff (portable electromyography device) while sleeping at home. The prevalence of SB was assessed by the Bruxmeter software, where SB was evaluated as “absent” (score zero), “light” (<2 episodes), “moderate” (between 2 and 4 episodes), or “severe” (>4 episodes). The prevalence was expressed with descriptive statistics in percentage using the number of detected cases out of the total number of patients.

**Results:** Eighteen patients (78.26%) were diagnosed with SB. The severity of SB was 55.5%, 5.5%, and 39% for the light, moderate, and severe scores, respectively.

**Conclusions:** Almost 8 out of 10 edentulous patients have SB. Approximately half of the SB-detected patients showed moderate to severe severity of bruxism as measured by a commercial, at-home device.

**Summary**

• Bruxoff is a portable holder that records masseter muscle and heart activity.
• The prevalence of sleep bruxism (SB) among edentulous patients is high.
• Almost half of the patients detected with SB showed moderate-to-severe severity of bruxism.
• Developing a more specific and sensitive tool for edentulous patients to detect SB is pivotal.

## 1. Introduction

According to the 2013 consensus, Lobbezoo et al. [1] described bruxism as a repetitive masticatory muscle activity (RMMA), which includes the clenching or grinding of teeth and/or the bracing or thrusting of the mandible. This definition reflects the current understanding that the central nervous system primarily governs bruxism [2]. This is especially significant for edentulous patients, who exhibit activation patterns of the mandibular muscles similar to those seen in dentate individuals [1]. The bracing or thrusting involves a force that moves the jaw forward or laterally, and these actions do not require teeth to make contact. As a result, edentulous patients can experience bruxism to the same degree as those with teeth [2].

In 2018, the same research group revised the consensus, proposing a new definition for sleep bruxism (SB) as masticatory muscle activity during sleep, which can be either rhythmic (phasic) or nonrhythmic (tonic). SB should be viewed not as a disorder but as a behavior that may serve as a risk (or protective) factor for specific clinical outcomes in otherwise healthy individuals [2]. Teeth grinding is generally practiced by the population and is, therefore, considered standard [3]. However, grinding becomes problematic when its signs and symptoms are severe, potentially leading to orofacial pain, including temporomandibular disorders (TMDs) [4]. TMD encompasses a range of musculoskeletal and neuromuscular conditions involving the masticatory muscles, the temporomandibular joint (TMJ), and their associated structures [5]. The prevalence of TMD in elderly patients requires further study, but it appears to be significant [6, 7]. Additionally, there is a potential link between Parkinson's disease and TMD [8].

In addition, other important risk factors associated with SB are gastroesophageal reflux disease (GERD), genetic polymorphism, as well as alcohol, caffeine, tobacco, and drug intake [9, 10]. SB has an underestimated and imprecise prevalence of an average of 12.8% in the adult population, with no gender differences [11, 12].

Furthermore, the last consensus proposed a grading system for SB as follows: (1) Possible SB is based on a positive self-report only; (2) probable SB is based on a positive clinical inspection, with or without a positive self-report; and (3) definitive SB is based on a positive instrumental assessment, with or without a positive self-report and a positive clinical inspection [2]. Instrumental methods for evaluating SB include polysomnography (PSG), which is regarded as the gold standard for SB detection [1, 13, 14]. However, it has some limitations, including that the examination is not conducted in the patient's home, which may not accurately represent their typical behavior in a natural environment. Additionally, the exam is associated with a high cost.

Portable electromyographic (EMG) devices have been suggested to solve these deficiencies partially, allowing patients to perform the exam in a natural sleep environment [14, 15]. Bruxoff is a compact, portable device that simultaneously records heart rate and masseter muscle activity. It uses electrodes with advanced signal analysis capabilities to detect EMG signals from the surface of the masseter muscle. Compared to PSG, Bruxoff has demonstrated sensitivity and specificity of 85% and 92%, respectively [14, 16]. While highly sensitive and specific tools are commonly used to detect SB in dentate patients [17, 18], to our knowledge, there are no studies in the literature that examine its use in edentulous patients. The study of bruxism in edentulous patients gained strength, especially after the bruxism consensus [2], and continues to be an essential area of research and clinical practice for several reasons. It is suggested that even in the absence of teeth, edentulous patients can still present parafunctional activity with the muscles of mastication [2]. Bruxism in edentulous patients can cause damage to soft tissues, such as the oral mucosa, and affect the stability and comfort of dental prostheses, leading to discomfort, inflammation, and even prosthesis failures [19]. This can significantly impact the quality of life and functionality of these patients. Furthermore, treating these patients can be complex and requires a multidisciplinary approach. Understanding how SB manifests in edentulous patients can provide insights into these interactions and potential integrated treatment approaches.

Thus, the objectives of this study were to assess, with a portable device, the prevalence and the severity of SB in edentulous patients' wearers of conventional complete dentures (CCDs).

## 2. Materials and Methods

### 2.1. Study Design

This cross-sectional study was reported using the report guide statement “Strengthening the Reporting of Observational Studies in Epidemiology (STROBE)” [20]. The Human Research Ethics Committee approved the development of this study under number 1.452.492. The study was conducted in accordance with the Helsinki guidelines [21].

### 2.2. Setting, Participants, and Study Size

#### 2.2.1. Eligibility Criteria

The convenience sample consisted of consecutive patients admitted to the Department of Dentistry of the Federal University of Santa Catarina, Florianopolis, Brazil, between 2017 and 2019 and met the eligibility criteria to participate. The patients sought care to change their old CCDs for new ones. Patients who met the eligibility criteria signed a written consent form.

#### 2.2.2. Inclusion Criteria

Fully edentulous adults from 40 to 75 years old who were unsatisfied with their old CCDs were included.

#### 2.2.3. Exclusion Criteria

Subjects under medical treatment, with daily ingestion of drugs acting on the central nervous system, or with neurological problems or movement disorders were excluded. Subjects who did not feel empowered to use the device Bruxoff were also excluded.

### 2.3. Variables, Data Sources, and Measurement

#### 2.3.1. Clinical Procedures: Fabrication of the CCDs

Patients who qualified for the study began treatment by replacing their old CCDs with new ones. During fabrication and installation, the CCDs were adjusted to achieve bilateral balanced occlusion. Patients were monitored for at least 1 month after receiving any necessary basal, and occlusal adjustments until the CCDs were functional and comfortable.

#### 2.3.2. Prevalence and Severity of SB: The Bruxoff Portable EMG Device

**Figure 1:**
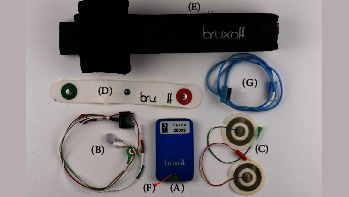
The Bruxoff device (A), the electrodes connection cable (B), the pair of electromyographical electrodes (C), the cardiac electrodes (D), the chest strap (E), the start/stop button (F), and the USB cable (G). USB, universal serial bus.

Patients were instructed to undergo the SB examination using the Bruxoff device. They were provided with a kit containing all necessary components for the test, including the user's manual (Figure 1). In addition, patients received instructions on using the device through a demonstration and a brief video recorded by the investigators. On the examination day, each patient was asked to wear the device and maxillary and mandibular CCDs while sleeping in their homes.

The Bruxmeter software (Bruxoff, Italy) generated a file containing data on the duration of the examination and bruxism detection. This information included the total number of bruxism episodes, the number of episodes per hour, the average heart rate, and the total number of masseter muscle contractions, categorized as phasic, tonic, or mixed contractions. For determining the prevalence of SB using the Bruxoff device, participants diagnosed with light, moderate, or severe SB were classified as “positive” for SB. At the same time, those with a “zero” score were considered “negative” for SB. The number of teeth-clenching episodes per hour was recorded. Each patient was assigned an SB score based on the frequency: “absent” (score zero), “light” (<2 episodes), “moderate” (2 to 4 episodes), or “severe” (>4 episodes) as recommended by the Bruxoff manufacturer. The results were analyzed by an investigator blinded to the patients' training in using Bruxoff.

### 2.4. Bias

To avoid possible biases in the study, study participants were given detailed explanations about using the device. A single trained researcher evaluated results and data completion.

### 2.5. Statistical Methods

The prevalence of SB assessed with the Bruxoff device was expressed with descriptive statistics in percentage utilizing the number of diagnosed cases out of the total number of patients. Means and standard deviations of the Bruxoff continuous variables were calculated using Microsoft Excel.

## 3. Results

### 3.1. Participants, Descriptive, and Data Outcome

Twenty-nine patients enrolled in the study began treatment with new CCDs. However, six (20.7%) of these patients could not complete the Bruxoff examination due to technical difficulties with the device. Data from the remaining 23 patients (13 females, mean age: 65 ± 2 years; 10 males, mean age: 66 ± 2 years) were analyzed.

### 3.2. Main Results

#### 3.2.1. The Bruxoff Portable EMG Device: Prevalence and Severity of Definitive SB

The full results of the examination with the Bruxoff device can be seen in Table 1.

The average duration of the sleep examination was 6 h and 56 min, with a standard deviation of 1 h and 46 min. The avarage number of total number of bruxism episodes was 11.91 ± 11.34, while the average number of episodes per hour was 2.36 ± 2.58. Eighteen patients (78.26%) out of 23 patients were positively detected with SB, whereas the other five patients (21.74%) were not detected with SB.

The distribution of the severity of cases using the Bruxoff device was also evaluated. In total, 55.5% (10 out of 18) had a light score, whereas 5.5% (1 out of 18) presented a moderate score, and, finally, 39% (7 of 18) of patients presented a severe score of SB.

## 4. Discussion

The results of our study suggest a high prevalence of SB in complete denture wearers. To our knowledge, very few studies assessed SB among edentulous patients [22–24]. This scientific gap is reinforced by the inherent difficulty in conducting longitudinal clinical studies evaluating the incidence and severity of bruxism [25].

According to the Consensus presented by Lobbezoo et al. [1], edentulous patients show the same activation patterns of the muscles involved in the mandibular movements as dentate patients. This was clinically confirmed with edentulous patients' wearers of maxillary CCDs and mandibular implant-supported prostheses by Mattia et al. [23], who reported that SB could occur with or without direct contact between the teeth, characterized by increased rhythmic masticatory muscle activity. Similarly, our study, using the Bruxoff device, observed a high prevalence of SB (78%) among CCD wearers. Moreover, when focusing solely on the positive SB cases identified by the portable device, nearly half of the sample (44.4%) exhibited clinically concerning severities (moderate to severe).

The study by Von Gonten and Rugh [24] from 40 years ago explored using a portable electromyography device to analyze the nocturnal muscle activity in four edentulous patients wearing or not wearing the complete denture. They concluded that the presence of dentures may reduce masseter muscle activity of the sleeping edentulous patient, regardless of the frequent use of the prosthesis during sleep, unlike the present study's findings, where even when using new dentures, the sample showed a high prevalence of rhythmic muscle activity during sleep [24].

The study by Mercado and Fulkner [22] also found a high prevalence of parafunctional habits in edentulous patients wearing complete dentures. Out of the 201 participants, 33.83% reported clenching or grinding [22].

Regarding the epidemiology of bruxism, the prevalence shows variability. A systematic review found a prevalence of 12.8% ± 3.1%, with the most included studies being questionnaire-based [11]. In contrast, one study found an average prevalence of 7.4% in a study detecting bruxism using the definitive method (PSG) in a Brazilian population [26]. This prevalence was much lower than in the present study, where the detection method was a portable electromyography. The advantage of portable electromyography is that the patient is in his/her usual environment for the test, with no interference from external environments. The current literature has shown high accuracy in detecting bruxism using these devices [27–29].

Although the study by Manfredini et al. [11] described decreased bruxism activities with increased patient age, recent studies have shown the opposite. Our study showed a high prevalence of bruxism in elderly patients, and the prevalence of TMDs that could occur as a result of bruxism has also been shown to be high for the same age group [6, 30]. Likewise, some elderly patients usually associate symptoms like pain as the consequences of older age, considering them as a normal condition and therefore pushing toward an underestimation of the diagnosis [6, 31]. In addition to the known clinical consequences in edentulous patients (muscle and joint pain, etc.), bruxism can also cause greater wear of the prosthetic teeth, and, as a result, the occlusal scheme achieved at the time of installation of the prostheses can be lost, causing a decrease in patients' chewing capacity. Furthermore, occlusal trauma transmitted to the alveolar crests can result in more pronounced resorption, increasing instability, and loss of prosthesis retention.

New studies including this age group of the population are necessary, and information regarding the condition and its possible consequences should be part of the rehabilitation treatments of these patients.

Detecting SB presents a significant clinical challenge, as many patients may be unaware of their teeth clenching or mandibular movements or lack a sleeping partner to confirm the behavior. Additionally, physical examination is not always definitive, as tooth wear does not necessarily indicate current bruxism activity. Furthermore, the existing clinical detection tools often need to be more conclusive. While clinical signs, symptoms, and self-report questionnaires are commonly used to identify SB, they frequently do not align with definitive detection methods for SB [32]. Stuginski-Barbosa et al. [33] compared the third edition of the International Classification of Sleep Disorders (ICSD-3) criteria for SB detection with the results of the gold standard PSG examination, and the results showed that the ICSD-3 diagnostic criteria items for SB had fair to moderate agreement with the PSG. Lastly, bruxism seems to be a transitory condition—patients do not clench their teeth all the time.

The Bruxoff device was selected for detection due to its advantages, such as lower cost than PSG and the convenience of allowing patients to undergo the examination in their sleeping environment [34]. This setup provides a more natural sleep experience than PSG, requiring patients to sleep in a laboratory. Additionally, Bruxoff offers high specificity and sensitivity relative to PSG [14]. However, it does have some drawbacks, including the absence of a monitoring system during use, which may conceal potential errors and necessitate retesting. Another limitation related to Bruxoff is that it has no audio and video recording, preventing recording any sound or visual activity (e.g., grinding). Furthermore, due to the lack of monitoring of the electroencephalogram, the device cannot actually record patients' sleep duration, as there is no possibility of knowing when the patient falls asleep and wakes up. Finally, the fact that the exam was performed for only one night may be a flaw, as the repeatability of the results could not be assessed [18].

Despite its innovative design, the Bruxoff EMG portable device has limited scientific evidence supporting its use, especially in edentulous patients. Given the initial technical challenges encountered with the device, this study can be seen as pioneering in applying the Bruxoff device for edentulous patients who wear CCDs.

Out of the 29 patients who received treatment with CCDs, six (20.7%) could not complete the Bruxoff examination due to various factors. Initially, the researchers encountered technical difficulties with the new device as part of the learning curve. Additionally, the device presented some issues, such as data recording failures. Technical support from the company took longer than expected to address these problems. Moreover, patients faced challenges in using the device correctly. Despite efforts to provide comprehensive education on proper use, many patients, mainly due to their advanced age, struggled with the device alone at home, even though they reported understanding the instructions.

The device's reliance on the patient's understanding and cooperation may limit its applicability, especially among elderly individuals who are less familiar with technology. This limitation is supported by a recent study by Thymi et al. [25], which reported a low enrollment rate for patients with SB using multiple single-channel EMG recordings, with only 11 out of the 98 planned patients participating. This prospective cohort study intends to explore the association between SB and (peri-)implant complications and challenges, including insufficient participant recruitment and failed EMG recordings. The small sample size hindered the ability to address the study's primary objectives and was primarily attributed to the complexity of the study protocol. EMG recording failures were due to poor signal quality and electrode detachment.

As strengths of this study, we highlight the innovative character of using a device considered a definitive method for detecting SB nowadays. In addition, the investigation of a population that is not primarily studied for this type of outcome. Also, we highlight the clinical importance of the detection and guidance of this condition for patients undergoing any prosthetic rehabilitation. Lastly, it is pivotal and strongly recommended that a detection tool for possible SB, such as a validated modified questionnaire, be developed to be more specific and sensitive to edentulous patients. As limitations, we can mention the small sample and the difficulty presented by some participants in using the device; however, it is completely understandable by the age group included in the present research.

It is also important to mention that despite methodological difficulties in defining secondary bruxism [35], some disease fields [36, 37], coffee consumption, smoking, other drugs [9], and medication [38, 39], can be associated with bruxism. In contrast, other variables, such as obstructive apnea, remain inconclusive [40]. These variables were not addressed in this study.

Despite the limitations, the study has internal validity due to the relative absence of systematic errors. As for the external validity, these results can be applied to a similar population, that is, elderly patients who are wearers of CCDs referred for treatment in a public university. More clinical studies with larger sample sizes, different types of prosthetic restorations, and more extended follow-up periods assessing bruxism in edentulous patients are necessary. Furthermore, using digital tools for diagnostic purposes should be better investigated.

## 5. Conclusion

The prevalence of SB among CCD wearers is high. Almost half of the patients detected with SB showed a moderate to severe degree of severity of bruxism.

## Figures and Tables

**Table 1:** Results of the examination with the Bruxoff device.

Patient number	Severity	Exam duration	Total bruxism episodes	Mean heart rate	Number of episodes/hour	Masseter contraction	Phasic contraction	Tonic contraction	Mixed contraction
1	Light	11:00	11	55	1	68	0	0	0
2	Light	09:10	18	62	2	623	116 (4)	25 (0)	0 (0)
3	Light	09:00	12	69	1.4	128	21 (2)	57 (6)	6 (1)
4	Severe	07:00	28	71	4.1	242	100 (8)	63 (11)	15 (0)
5	Severe	04:52	26	46	5.5	52	6 (3)	6 (2)	0 (0)
6	Severe	07:00	12	79	4.1	262	112 (7)	59 (1)	20 (0)
7	Negative	07:17	0	70	0	126	7 (0)	3 (0)	0 (0)
8	Light	08:01	8	69	1.1	383	91 (2)	16 (0)	9 (0)
9	Light	08:00	3	64	0.4	233	0 (0)	0 (0)	0 (0)
10	Negative	06:00	0	73	0	34	9 (0)	4 (0)	2 (0)
11	Light	07:00	7	68	1	285	74 (2)	35 (2)	20 (2)
12	Light	06:00	1	63	0.3	192	0 (0)	0 (0)	0 (0)
13	Negative	08:25	0	50	0	254	131 (0)	47 (0)	12 (0)
14	Moderate	07:00	20	73	2.9	40	21 (11)	1 (0)	0 (0)
15	Severe	04:38	25	58	6	115	11 (2)	12 (3)	0 (0)
16	Severe	02:50	13	63	5.6	24	0 (0)	0 (0)	0 (0)
17	Negative	07:24	0	60	0	144	58 (0)	36 (0)	2 (0)
18	Severe	07:05	26	64	7	159	0 (0)	0 (0)	0 (0)
19	Light	06:00	3	62	0.6	76	0 (0)	0 (0)	0 (0)
20	Negative	05:00	0	62	0	74	0 (0)	0 (0)	0 (0)
21	Light	07:52	7	62	0.9	96	25 (3)	36 (2)	16 (1)
22	Light	08:00	13	65	1.9	124	46 (3)	8 (2)	0 (0)
23	Severe	05:00	41	72	8.5	175	0 (0)	0 (0)	0 (0)
	Mean	06:56	11.91	64.35	2.36	169.96	—	—	—
	Standard deviation	01:46	11.34	7.60	2.58	135.38	—	—	—

## Data Availability

Additional data from this article will be shared upon request from the corresponding author.
